# Healthcare navigation services support access to gender-affirming care: A qualitative analysis of chat conversations

**DOI:** 10.1371/journal.pone.0333168

**Published:** 2025-10-23

**Authors:** Patrina Sexton Topper, Seul Ki Choi, Arianna Hall-Grix, Enmanuel Minaya Fernandez, Jaclyn Marshall

**Affiliations:** 1 Villanova University, Fiztpatrick College of Nursing, Villanova, Pennsylvania, United States of America; 2 Eidos LGBTQ+ Health Initiative, Department of Family and Community Health, University of Pennsylvania, Philadelphia, Pennsylvania, United States of America; 3 Included Health, San Francisco, California, United States of America; Autonomous University of Madrid: Universidad Autonoma de Madrid, SPAIN

## Abstract

**Background:**

As transgender and gender diverse (TGD) people become increasingly visible, healthcare solutions in the United States have also expanded to meet their needs. As TGD people and their families pursue gender affirming care (GAC), they also seek support to navigate complex healthcare and insurance systems. With increased demand for GAC, virtual healthcare navigation services in the U.S. have developed tailored approaches to address gaps in systems for this population.

**Purpose:**

The purpose of this study was to qualitatively explore the role and influence of trained care coordinators (CCs) from a virtual navigation service in meeting commercially-insured TGD people’s GAC needs.

**Methods:**

We examined and analyzed redacted chat conversations between CCs from a virtual healthcare navigation service in the U.S. and members seeking assistance. Reflexive thematic analysis of the data set involved multiple rounds of coding and reflexive analytic processes to develop themes.

**Findings:**

We developed three intersecting themes: 1) *Provide Dignified Experiences*, 2) *Promote Affirming Patient-Provider Relationships*, and 3) *Navigate Complex Insurance Systems*. Sub-themes associated with each of the themes include: 1) Shared life experience; Exploring gender identity and GAC options in safe spaces; 2) Connections with vetted providers; 3) Access accurate information about insurance policies; Assistance with appeals process; and Obtaining letters of support.

**Discussion/Conclusion:**

Navigating U.S. healthcare and insurance systems presents unique challenges for TGD people. While challenges to accessing care are well documented, our findings suggest that healthcare navigation services supported by trained CCs substantively assist TGD patients overcome obstacles. Our findings revealed multiple levels at which the service provides benefits -- individual, interpersonal, and structural. These levels of influence have clinical and policy implications for improved care for TGD people. Interventions like tailored, virtual healthcare navigation services are scalable and should be evaluated for reach and magnitude of impact beyond commercially-insured populations.

## Introduction

Across populations and care settings, navigating complex, fragmented healthcare systems have led to health disparities in the United States. Experiences of stigma, discrimination, and unequal treatment among marginalized populations often exacerbate these challenges [1,2]. Transgender and gender diverse (TGD) populations, whose gender identities differ from their sex assigned at birth, traverse unique complexities as they attempt to meet their health and social care needs within healthcare settings not designed for them [3]. Often, TGD people seek out gender-affirming and inclusive care. Yet, in the United States, finding those settings proves challenging for many [4] with recent policies introducing re-imagined barriers to care. Further, navigating complex health systems and insurance policies, with added layers of stigma and discrimination, can lead to healthcare avoidance, which contributes to health disparities among TGD populations [3,5,6]. Compounding issues related to navigating complex systems, providers often lack the knowledge and training to competently treat TGD individuals, which may introduce confusion and inefficiencies into already complex healthcare situations [7–9].

Gender-affirming care (GAC) may include legal, social, psychological, behavioral, and medical interventions that support TGD individuals in affirming their gender identities [3,5,6]. GAC is ideally patient-centered, holistic, interdisciplinary, well-coordinated, and respectful care for TGD people across the lifespan [3]. Evidence suggests that access to GAC promotes positive mental and physical health [7]. For example, gender-affirming hormone therapy (GAHT) and other medications, along with surgeries, may reduce gender dysphoria and comorbidities [8,9]; while social support and community connectedness benefit TGD [7].

TGD people and their families who encounter unique barriers to care may benefit from tailored assistance. Navigation services and care coordination often facilitate TGD patients’ efforts to utilize healthcare services and access the care they need [10]. Yet, little research has explored how and why TGD people and families may benefit from healthcare navigation services to meet their gender-affirming social and medical-surgical needs in the United States. Healthcare navigation care coordinators (CCs) may provide a scalable intervention that reduces gaps in care and improves quality for vulnerable groups like TGD people. Therefore, the objective of this study was to qualitatively explore the role and influence of trained CCs from a U.S.-based virtual healthcare navigation service on commercially insured members’ access to GAC.

## Methods

We conducted a qualitative study of the redacted chat conversations between members and CCs from a U.S.-based virtual healthcare navigation service, Included Health (IH). The study used Braun and Clarke’s approach to reflexive thematic analysis (RTA) [11,12], informed by a symbolic interactionist (SI) theoretical orientation [13,14] to address our research question. We selected RTA as an appropriate methodological approach due to its theoretical flexibility and suitability for analyzing complex phenomena. RTA allows for combined deductive and inductive analysis, without dependence on *a priori* coding frames. This seemed especially important for exploring largely understudied interactions between CCs and commercially insured TGD. SI, which is concerned with meaning making and social order through social interaction [13,14], grounded this interpretivist, constructivist exploration of CC and member interactions that dealt with access to GAC in complex environments.

### Ethical and consent statement

This study used deidentified data from a virtual chat platform, ensuring that the authors did not have access to any identifiable information about the members. Members consented to have their deidentified data used for research as part of the registration and enrollment process for the IH healthcare navigation and virtual care service. This consent occurred when they agreed to the data privacy policy [15]. Members documented consent to their data contributing to research, in an electronic format. No minors were involved in this study. Data were deidentified which ensured that the authors did not have access to any identifiable information about members. Compensation was not provided for study participation and minors were not involved in research. The University of Pennsylvania Institutional Review Board (IRB) reviewed the study’s protocol and concluded it was exempt as it did not meet the regulatory definition of human subjects research and, therefore, did not require formal IRB review. This study follows the Consolidated Criteria for Reporting Qualitative Research (COREQ) reporting guidelines.

### Study sample and data

We conducted RTA [11,12] of the redacted chat conversations between members and CCs from a U.S.-based digital health application that includes virtual navigation and healthcare delivery, IH. Chat conversations took place between July 1, 2022, and June 29, 2023. IH provides healthcare navigation and virtual care services as an employer-provided benefit to employees and their dependents (“members”) and by health plans for their insured members, including Medicaid, Medicare, and commercially-insured members [16]. Healthcare navigation services, including claims advocacy, high-quality provider recommendations, virtual second opinions, and general care navigation assistance, are available via self-service, in-app messaging, or live telephonic support with the care coordinator team. Virtual care is also available and delivered via video. With the exception of virtual care visits being subject to health plan cost-sharing, members do not need to pay to use the IH virtual services [16]. IH also delivers concierge advocacy and navigation services tailored to the healthcare needs of sexual and gender minorities (SGM), (‘members’), through the LGBTQ+ Health Offering (‘LGBTQ+ Navigation’). Members may receive support across four areas: 1) access to specialized CCs; 2) connections to vetted in-network providers; 3) assistance navigating benefits focused on LGBTQ+ health needs; and 4) education and advocacy for clinical and non-clinical needs. Within the navigation service, in addition to telephone and email communications, members can engage with CCs through a text chat function to address questions and concerns. Chats among the TGD patient population offer an innovative data source to explore access to GAC.

The LGBTQ+ Navigation service employs a rigorous vetting process to ensure the over 20,000 providers included in their proprietary directory deliver culturally humble and inclusive care for LGBTQ+ members. CCs, who bring lived experience, are trained in investigative interviewing and gather information by speaking directly to providers and their staff; member feedback is also gathered post-visit to keep the directory up to date. In addition, CCs complete a 4–5-week training program covering benefits navigation, social determinants of health, family building, common LGBTQ+ health concerns, patient advocacy, and intuitive listening [15].

Data mining health-related chat conversations can lead to novel insights about care accessibility [17] and generate meaningful research questions [18] about how to reduce gaps in care that may lessen health disparities in this population [19]. The study sample initially included 1,353 chat records between 469 unique members and CCs among those who had access to the LGBTQ+ Navigation offering. Due to time and resources constraints, the 1,353 chats were reduced to 660 chat records; the process by which we reduced the data set to 660 chat is described below.

#### Intake data.

Before members and CCs connected through the IH chat function, members submitted service requests. Request forms enabled members to identify the type of support they desired (e.g., provider request, information, support group, insurance benefits questions) and optional fields for race, ethnicity, sexual orientation, and gender identity (SOGI). Members submitted a single service request for each unique need, such as identifying an affirming provider (e.g., endocrinologist, psychiatrist) or support (e.g., insurance benefits navigation, pre-authorizations for surgical procedures). Each service request constituted a *case*. Thus, some members had multiple *cases* open simultaneously to navigate different care and information needs.

#### Chat data.

Service requests associated with the LGBTQ+ Navigation offering filtered down to a team of CCs consisting of a diverse group of self-identified LGBTQ+ people and allies of the LGBTQ+ community, with intimate knowledge of the challenges and barriers the community faces in both day-to-day life and seeking quality care. Text chat conversations from LGBTQ+ Navigation were aggregated and redacted. Only chat conversations among members whose employers opted into the healthcare company’s data agreement were included. The app-based chat conversations are securely stored by IH. Redactions were completed with a proprietary algorithm to remove protected personal information (PPI) from text chat records before sharing data with the research team. Redacted chat records consisted of text exchanges between a member and CC from a specific time. Members and CCs sometimes had multiple chat records pertaining to a single or multiple service requests and exchanged messages at different times.

From a total data set of 13,940 chat records related to 3,340 unique service requests among 2,136 members with the LGBTQ+ Navigation benefit through IH, we identified 1,353 chat records related to 849 service requests for 469 unique members seeking GAC. We defined GAC broadly to include domains such as biomedical interventions, informational resources, support services (e.g., mental health support, WPATH (World Professional Association for Transgender Health) letters of support), or legal assistance. We identified the subsample of 1,353 chats by applying key TGD-related search terms (e.g., gender dysphoria, gender identity, gender affirmation, transgender, non-binary, deadname), terms related to gender-affirming medical and surgical treatments (GAMST) (e.g., “top surgery,” “bottom surgery,” gender-affirming hormone therapy, endocrinologist), or GAC broadly (e.g., laser hair removal, “legal name change”, “affirming provider”, gender affirming counseling,) to filters using Microsoft Excel. Records were filtered in Excel and manually reviewed to ascertain relevance to TGD lives and GAC. Records of questionable relevance were reviewed through a triangulation process that included evaluation of other chats with the same member and their intake data. If the team determined a chat record did not relate to GAC, it was removed from the data corpus (e.g., if a chat related to a cisgender, gay male’s request for a primary care doctor, or a cisgender lesbian’s inquiry about fertility interventions). After reading and reviewing 1,353 records in the step of data familiarization [20] to understand the scope and content of the data set, a further step of reduction was undertaken for more detailed coding procedures within the analytic team.

### Data analysis

All relevant records were uploaded to Dedoose v.9.0.107 for RTA [21]. We employed inductive and deductive analysis, evaluating both manifest and latent content. Data familiarization of 1,353 chats included two analysts (PST and AH) reading chats to get a sense of content and scope of interactions between members and CCs through the chat function. Next, three analysts (PST, EMF, and AH) drew on a subset of chats to conduct an initial round of inductive coding. They then constructed a preliminary codebook using *in vivo* codes and key terms used in the data reduction phase (see above) to guide later cycles of line-by-line coding [22]. Due to time and resource constraints, a random subset of 660 chats from 1,353 records were selected for line-by-line coding, using a random number generator. The 660 chats were coded by four analysts (PST, SKC, EMF, AH) using the preliminary codebook and inductive codes added to the codebook throughout analysis. After all chats were coded, codes that had similar definitions were collapsed into a single code. Codes were classified and compared to detect differences and similarities [20,23], and data collated by code were examined. The team abstracted collections of codes into broader categories, assigned descriptive labels to code clusters, and developed themes iteratively using thematic mapping techniques [20,22]. In each of these processes, the team consulted data to assess fidelity. The team then prepared brief summaries of themes.

Throughout the data familiarization and coding processes, analysts engaged in extensive memoing to document impressions, practiced reflexivity [12,24] through analytic memoing and team discussions, and tracked analytic decisions. Themes were constructed and defined iteratively until we reached thematic sufficiency; meaning the categories and themes sufficiently accommodated data corpus [25]. We then stabilized theme definitions. Descriptive statistics were used to summarize the study participants’ characteristics in SAS software version 9.4 [26].

### Rigor

RTA foregrounds the positionality of the research team across the research design and analytic process [12,24,27]. While theoretically flexible, RTA requires sustained reflexive processes with respect to the research question and analytic processes throughout the project. According to Olmos-Vega and colleagues [12], reflexivity involves “a set of continuous, collaborative, and multifaceted practices through which researchers self-consciously critique, appraise, and evaluate how their subjectivity and context influence the research processes” (p. 242). Our team enacted these processes by acknowledging personal experience and social positions relative to access to care, privileges, blind spots, and biases related to interpreting care navigation. The team consisted of individuals from different racial and ethnic backgrounds, ages, sex and gender identities, levels of research or clinical experience, and identified as SGM ourselves or were allies of the community. Analysts hold advanced credentials that include BSN, MPH, MS, and PhD degrees. We recognize our shared commitment to health equity and advocacy for SGM groups who experience stigma or discrimination across social, structural, and healthcare continua, which shaped our perspectives. Analysts wrote positionality statements that were revisited throughout analysis, and the team met regularly to discuss coding and thematic development. Discussion integrated reflections informed by life experiences and subjectivities [12,28]. Rather than try to void our subjectivities, we engaged them as resources. Ongoing reflexive practice promoted credibility and authenticity of findings. Furthermore, we enacted the following processes to enhance trustworthiness and transferability: maintained an audit trail, memoed analytic decisions, collectively examined cases that deviated from patterns in the data, and engaged in multiple coding cycles [29]. Selected chats (10% of the total) were checked for coding agreement across coders, and disagreements were reconciled through robust discussion within the team. As mentioned above, the team consisted of people from diverse backgrounds and experiences, all of whom contributed rich and nuanced perspectives integrated into the analytic process and reflected in the development of themes described below.

## Findings

### Study sample characteristics

A summary of the study sample demographic characteristics is found in Table 1. The study sample of 469 members includes TGD individuals or parents and guardians who engaged with the service on behalf of their TGD children. The sample represents a broad range of individuals across race and ethnicities, SOGI, and census region. Members lived in all four census regions, ranging from 32% in the West to 19% in the Midwest and Northeast. Most members identified as White (69%), followed by 11% Hispanic, 10% Black or African American and 7% Asian. The sample also represents a range of self-selected gender identities. Approximately 80% of the sample identified as TGD (25% transman, 26% transwoman, 0.3% transgender, 24% non-binary or non-conforming, 4% other, and 2% agender). Almost 19% identified as a man or woman. The most prevalent sexual orientation was queer (36%), followed by bisexual (21%), lesbian (18%), gay (13%), and heterosexual (12%). Almost 96% of the LGBTQ+ Navigation services were provider requests; the remaining 4% were requests for support or resources.

**Table 1:** Types of service requests and demographic characteristics of members who used the LGBTQ+ Navigation service between July 1, 2022, and June 29, 2023.

	N (%)
*Service Request (n=849)*	
**Types of Service**	
Provider Request	813 (95.76%)
Resources/Support	36 (4.24%)
*Member Characteristics (n=469)*	
Race and Ethnicity* (n=276)	
Hispanic	29 (10.51%)
American Indian or Alaskan Native	3 (1.09%)
Asian	20 (7.27%)
White	191 (69.20%)
African American	28 (10.14%)
Hawaiian or Pacific Islander	1 (0.36%)
Other	2 (0.72%)
Not Applicable	13 (4.71%)
Pronouns* (n=275)	
He/Him/His	117 (42.55%)
She/Her/Hers	128 (46.55%)
They/Them/Theirs	84 (30.55%)
Ze/Zir/Zirs	1 (0.36%)
Not Applicable	5 (1.82%)
Gender Identity*† **(n=342)**	
Man	29 (8.48%)
Woman	36 (10.53%)
Transman	84 (24.56%)
Transwoman	89 (26.02%)
Transgender	1 (0.29%)
Agender	7 (2.05%)
Non-binary non-conforming	80 (23.39%)
Other	14 (4.09%)
Not Applicable	11 (3.22%)
Sexual Orientation*† **(n=352)**	
Heterosexual	42 (11.93%)
Gay	47 (13.35%)
Lesbian	65 (18.47%)
Bisexual	75 (21.31%)
Queer	125 (35.51%)
Asexual	21 (5.97%)
Pansexual	66 (18.75%)
Aromantic	4 (1.14%)
Other	20 (5.68%)
Not Applicable	28 (7.95%)
**Region (n=469)**	
Midwest	90 (19.19%)
Northeast	89 (18.98%)
South	137 (29.21%)
West	149 (31.77%)
More than two different regions	4 (0.85%)

*Non-mutually exclusive categories; participants were allowed to select all that apply. Not all members responded to race/ethnicity or SOGI items, which resulted in missing data. Ns reflect the number of members who provided a response to the optional demographic fields within the intake form.

†Categories reflect predetermined-identifiers and/or fill-in text options from original service selection and demographic intake data.

### Qualitative themes

**Fig 1:**
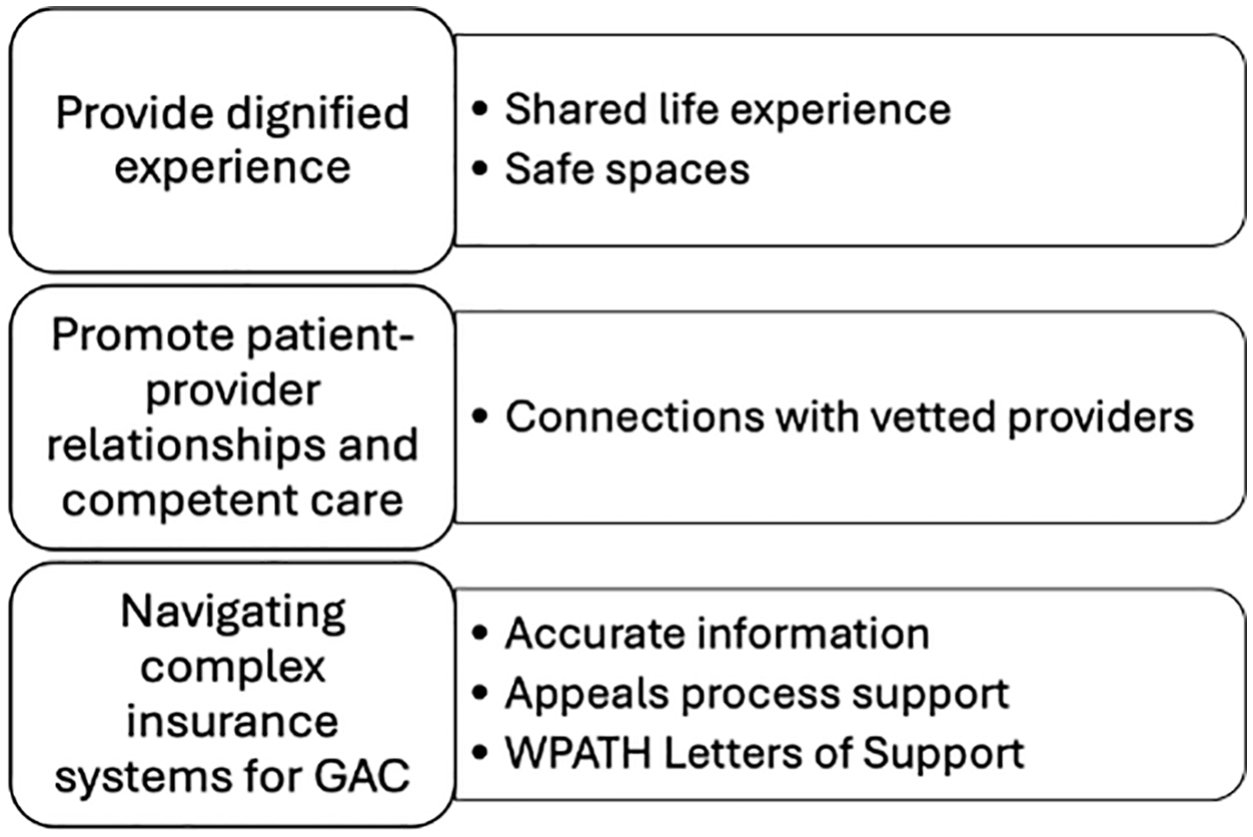
Themes and inter-related subthemes. Three themes and related sub-themes identified through RTA of chat conversations between members and LGBTQ+ Navigation care coordinators between July 1, 2022, and June 29, 2023.

Three overarching themes illuminate the roles and influences of CCs in helping TGD people access competent and affirming care. Although themes can be conceptualized as distinct, chat conversations provide evidence of their dynamism and interconnectedness--the themes impact and build on one another. In Fig 1, we present each theme and related sub-themes.

Table 2 displays themes, sub-themes, and illustrative quotes. Where possible, member characteristics, including state of residence, race and ethnicity, SOGI, and type of initial request submitted are provided with quoted chat content. Below, we explore themes and sub-themes in how each relates to the provision of innovative care solutions and support for this marginalized population that, in aggregate, faces health disparities.

**Table 2:** Summary of themes, sub-themes and illustrative quotes from chat conversations between members and LGBTQ+ Navigation care coordinators between July 1, 2022, and June 29, 2023.

Theme	Provide Dignified Experience		Promote Affirming Relationships and Competent Care		Navigate complex insurance systems	
**Sub-theme**	Shared life experience—no need to explain oneself	Exploring gender identity and GAC in safe spaces	Connections with vetted providers	Access to accurate insurance policy information	Appeals assistance	Obtaining letters of support
**Illustrative quotes**	Member: I’m looking for an OBGYN for a consult for laparoscopic hysterectomy for my [Partner/Spouse]. He’s transgender and would like to find a surgeon whose worked with trans guys before. … CC:... I’ll be glad to work on a search for a trans experienced OBGYN for you, [Name]. I know this was also important to me when I had my hysterectomy as a trans man. (NJ; Bisexual, Gay, Queer, Nonbinary/Gender Non-conforming; Non-Hispanic White; Provider Request)	Member: I have really been struggling with my gender dysphoria. I don’t necessarily want to transition; I do not identify as male. I consider myself more female/non-binary… I find myself especially depressed with my appearance …this is not how I want to live. CC: [Name] thank you for trusting me to share so openly with the struggles impacting you. I know that can be tough especially with someone you do not know well. …I am transgender and struggle … (AL; Lesbian/Genderfluid; Non-Hispanic, White; Provider Request)	Member: Since this will be the next step in gender-affirming surgery it would be good to have someone that has experience in this area. After this surgery there will be a plastic surgeon involved in final phase of surgeries. CC: That is very understandable! I will start vetting potential options that are experienced in gender-affirming care/hysterectomies and update you on my progress. (TX; Transgender Man, No Sexual Orientation Information; Non-Hispanic, White; Provider Request and Resource Support)	CC: I will research hair removal providers in your area, contact the providers to confirm that they are trans-affirming and create a comfortable environment for trans clients, and confirm when they are available for new clients. This can take a little bit of time … Member: Awesome. Thank you so much. I have been so overwhelmed with just finding out what’s covered and what not...that I just kinda have been at a standstill. (MO, Queer, Transgender Woman, Provider Requests, Non-Hispanic, White)	Member: Also, I just received the determination from [INSURER] on my appeal … and they denied it, citing the … pricing as the reason even though they are not in compliance with that term. CC: Thank you for sharing that update! I am disappointed to learn that [INSURER] denied your first appeal... Since appeals can be time sensitive, it would be helpful to submit this form sooner than later…After you submit your form, I will have authorization to call [INSURER] on your behalf…. (OR, No SOGI or Race/Ethnicity Demographic data Provider Requests--Plastic Surgeon/Endocrinology/Physical Therapy)	Member:... When I called to schedule I was told I needed a clearance letter before I could schedule but nobody gave me the criteria of the letter. … I have been trying to get on T for [time frame] and I’m still not clear on what my insurance covers/ requires…. I would love some help establishing if I have the right team qualification wise as well as what all I need to receive documentation wise to move forward. CC: I’m sorry you’ve been running into these obstacles... For [INSURER] … you’ll need the clearance letter from a provider that is equipped to treat gender concerns/gender dysphoria -- same as top surgery … (GA, NO SOGI or Race/Ethnicity Demographic Data, Provider Request—PCP)

#### Provide dignified experiences.

The overarching theme, *Provide Dignified Experiences*, captures a trend across chat conversations. CCs offered support services and demonstrated an effort to create dignified experiences for TGD members and families across the data corpus. Within and across chats, CCs made it clear that they aimed to knowledgeably treat members and member inquiries with respect and care. Two sub-themes help to further elaborate on and substantiate this theme: *Shared Life Experience/No Need to Explain* and *Explore Gender Identity and GAC in Safe Spaces.* Below, we examine these sub-themes in greater depth.

*Providing dignified experiences* to members involves the establishment of safety and trust through social interaction. We define safety as the conditions that contribute to a sense of security, harm prevention, and efforts to reduce the risks of exposure to experiences of discrimination and alienation in clinical settings. We define trust as confidence in the reliability and integrity of CCs and their support services. Establishing safe spaces within the context of chat conversations contributed to building and maintaining a sense of trust among members. CCs demonstrated knowledge about the circumstances or experiences members described and often conveyed shared life experiences. For example, throughout the chats, CCs echoed the sentiment, “I understand completely, and I want for everything to make you feel secure.” Conversations between CCs and members indicated trust and safety that promoted conditions to assist TGD individuals through complex social and clinical processes that often pose challenges for this population. CCs from the LGBTQ+ Navigation offering communicated that their service offering was tailored to meet the health and/or social needs of TGD through cues, words, and the structure and flow of services. Furthermore, CCs communicated to TGD people and their family members that they were trustworthy and their experiences credible--they prioritized and centered acknowledgement of their lived experiences and promoted safety of this population throughout chat conversations. Communication of shared life experiences and creating safe spaces in which members could honestly explore gender identity further marked how CCs provided dignified experiences in which TGD members and families pursued GAC with confidence and support.

Promoting dignified experience was sometimes unexpected and a pleasant surprise among members, as evidenced below. This exchange between a CC and member from Colorado highlights an interaction imbued with a sense of surprise and gratitude. Interactions between the member and the CC, the member and the organization (IH), and the member and (insurance) policies that accommodate her life experience as constructed in conversations reveal meaning the member has derived. In this case voice modification interventions suggest to her that others see her life and needs as worthy of support. Moreover, these symbols of the value of her life and lives like hers may “save lives”:

**Member:** Thank you so much. I didn’t have much luck when trying to research doctors so that’d be so helpful if you could find one. I’m not sure if [LOCATION] even has any surgeons who primarily work with LGBTQ + . …Thank you again so so much! When I see voice modification as well on that list, *I am so excited. I never thought I’d see benefits like this in my lifetime.* I wish my sister was still with us to see all these beautiful changes. If she just could’ve hung on. Services like this, the work you’re doing, it’s going to save lives.

**CC**: That is an incredible and heartbreaking thing to hear, … Being able to do this work is an honor and a privilege.

(CO; No SOGI or race/ethnicity data; Provider Request)

From the text we discerned that the member does not feel confident that in local providers offering competent and affirming care to meet their needs. In this chat the member seeks the help of the navigation service to see if the organization may have better luck. The conversations suggest that the member would prefer to work with providers who have experience with and even primarily treat the LGBTQ+ community. Further, the member acknowledges feeling “excited” by the option to access voice modification services to support gender alignment. The member states, “I never thought I’d see benefits like this in my lifetime,” calling both the benefits and services provided by IH (organization) and CC (interpersonal support) “beautiful changes.” This rich exchange highlights the innovation provided in this tailored navigation delivery.

#### Shared life experience--no need to explain.

Communications about *Shared life experience* further contributed to cultivating a sense of safety and trust between CCs and members. The illustrative quote in Table 2, presented here in its unabbreviated form for ease of reading, demonstrates this trend across chats:

**Member**: I’m looking for an OBGYN for a consult for laparoscopic hysterectomy for my [Partner/Spouse]. He’s transgender and would like to find a surgeon whose worked with trans guys before. [SPOUSE NAME] has had experiences with OBGYN providers in the past and is concerned about being turned down for treatment because some doctors have concerns about maintaining fertility.

**CC:** Thank you for this request. I’ll be glad to work on a search for a trans experienced OBGYN for you, [Name]. I know this was also important to me when I had my hysterectomy as a trans man. I consider it an honor to assist a sibling in Community.

(NJ; bisexual, gay, queer, non-binary/gender non-conforming; Non-Hispanic White; Provider Request)

Exemplary of other exchanges across the data set, this CC shares that he had the same surgical procedure that this member’s spouse wants to have. The CC understands the conditions of the request: namely, wanting to work with a provider who has experience with the surgical procedure itself within the target population, and who will not threaten to refuse care. The same conditions were important to him in his own search. Establishing the shared experience contributes to a sense of safety and trustworthiness in the CC to find a surgeon who is a good fit and advocate effectively for the member. Mutual understanding of the level of importance and nuances of finding someone to work with who will not introduce harm are conveyed in the brief exchange.

The shared life experiences communicated in chats indicated “no need to explain” some topics. Chat conversations reflected mutual understanding, which created opportunities for members to state relevant details about the help they needed. For example, this CC explained to a member:

**CC:** [t]rust me, I understand the frustration related to having to “prove” your trans after a lifetime of undoubtably knowing it yourself. The good news is that there are many, many providers who also understand this frustration who are willing to write a letter [of support]

(NY; No SOGI or race/ethnicity data; Provider Request)

This quote highlights the importance of understanding both the frustration and the experience of “proving” one’s gender identity to a third party who has the power to control access to important interventions. This subtheme gets at elements of social safety and professional trustworthiness. This CC communicates understanding with the member in the following excerpt:

**CC**: it’s totally understandable that you might go into things with that mindset--obviously being trans in our society can be really scary, … there’s nothing wrong about feeling nervous to see someone that you don’t know much about.

(GA; No SOGI or race/ethnicity data; Provider Request).

The CC reflects awareness of the fear associated with being “trans in our society”. This statement tells the member, “I get it” and your feelings are legitimate. Moreover, the CC communicates that symbolically healthcare providers can “be scary” “in our society” because there is legitimate reason to not trust them or the institutions they represent. Yet, because the CC and member establish the shared meaning, they can also collectively notice when a different process and organization help you an alternative--finding a provider who is safe and does not represent that which is untrustworthy. This alternative path may in turn foster conditions of safety.

#### Exploring gender identity and GAC options in safe spaces.

The open exploration of gender identity and potential interventions with CCs who provided members with respectful and affirming responses supports the broader theme of *Providing Dignified Experiences*. The unabbreviated chat exchange presented below (abbreviated in Table 2) exemplifies this connection. A member, who lives in Alabama and identifies as genderfluid, initially inquired about GAC options. The member confides about struggling with gender dysphoria and self-harm behaviors.

**Member**: I have really been struggling with my gender dysphoria. I don’t necessarily want to transition; I do not identify as male. I consider myself more female/non-binary. [Redacted] as a breast reduction, I find myself especially depressed with my appearance as well as experiencing back pain from the size and weight of them. This causes me to self-harm in the form of binge eating to the point of making myself sick, and I haven’t been able to get control of this and it’s causing me anxiety because this is not how I want to live.

**CC:** [Member Name] thank you for trusting me to share so openly with the struggles impacting you. I know that can be tough especially with someone you do not know well. Gender Dysphoria, like body dysphoria, can be difficult to manage. It isn’t uncommon for us to develop some less then optimal coping mechanisms when confronted with these feelings. You should be incredibly proud of yourself for taking this step to seek out the help you need to support the changes in your life you want to see. I am transgender and struggle with binge eating so know a little of how it feels to be on this journey.

(AL; lesbian/genderfluid; Non-Hispanic, White; Provider Request)

Though this member does not intend to start gender transition, the gender dysphoria experienced, and self-harm behaviors identified indicate a critical need for affirming and competent interventions. Moreover, the chat gives voice to critical needs to address the interconnected physical, mental, and behavioral health issues faced by many TGD people. Other members who confided in CCs about gender exploration requested additional resources or to connect with support groups highlighting the value attributed to social supports.

Parents or guardians of TGD children contacted CCs to determine 1) where to start when children disclosed or parents discovered a shift related to gender that warranted finding appropriate medical or social support, 2) what types of resources parents had access to in order to help children and families through processes of discovery and care, and 3) to connect parents with competent and affirming providers who would treat their children with respect. For example, this parent of an adolescent child who is both transgender and neurodivergent reported frustration with care from a recommended provider group:

**Member** we’ve received many services at [Hospital Name]. Our experience has been hit-or-miss in terms of competence – [Hospital Name] does not have pronouns or gender identity in their [redacted] despite my having advocated for this for [redacted], and does not ask patients this information, so my child is typically misgendered there.

(MA; non-binary/gender non-conforming, pansexual; Black/African American; Provider Request)

This parent who lives in Iowa explains the need for additional support from a healthcare navigation service, “to be completely transparent I am working my local network of moms groups and LGBTQ resources as well, but I need all the help I can get to find someone take care of [Name]” (IA; non-binary/Gender non-conforming, Non-Hispanic White and Other(unspecified); Provider Request). Parents often initiated chat conversations with CCs from positions of vulnerability or frustration because they did not necessarily know how best to support their children.

CCs provided safe spaces and trustworthy support with which members themselves, or parents of children could explore complex terrain associated with gender and access to competent, affirming care. In sum, CCs established and cultivated environments of safety and trust and created safe spaces in which members could explore dimensions of gender identity. Taken together, the larger theme of *Providing Dignified Experiences* to members with GAC needs took form.

#### Promote affirming patient-provider relationships and competent care.

The theme *Promoting affirming patient-provider relationships and competent care* represents the importance of the navigation services’ capacity to connect members with vetted providers. As reported in Table 1 above, provider requests accounted for most member-initiated contact with the healthcare navigation service. Members submitted service requests to find healthcare providers who a) were covered by their insurance benefits and b) came recommended for TGD patient populations.

#### Connections with vetted providers.

In addition, connections were made across the spectrum of affirming care services including vetted speech therapists, vocal training, hair removal specialists, mental health professionals, and support groups or community resources. Chats revealed a trend in discussions: CCs helping to link members to credible gender-affirming care providers and services (e.g., social workers and psychologists, primary care providers, surgeons, dentists, hair removal services). Making connections with evaluated providers reduced the labor (finding providers who deliver affirming and competent care) and risk (of going to a provider who lacks the capacity to adequately care for TGD patients or the introduction of rejection, stigmatization, discrimination, and alienation into a care encounter) for the members.

CCs tried to ensure that recommended providers met the organization’s quality criteria for their LGBTQ+ members. Chat interactions highlighted the critical importance of connections to and appointments with clinically competent, culturally sensitive, affirming providers and institutions for members. Chat exchanges showed vetting processes in two parallel modes: first, an organizationally constructed directory of vetted providers, and second, active engagement in concurrent vetting of additional providers who aligned with member preferences in particular specialties or geographic locations. The latter allowed for the expansion of the formal directory. In many cases, CCs made statements such as, “I can confirm they meet our vetting criteria, and we feel confident they are friendly and affirming.” Members expressed gratitude for the chance to work with competent and affirming providers from the IH directory in many instances. For example, this member replies encouragingly to an inquiry about their experience with an IH vetted provider:

**Member:** [T]he appointment went amazingly… she was the best and super-duper affirming... I was able to talk about my pronouns, my sexual identity, my gender identity, and my goals … I am absolutely rambling but to talk to someone who respected me as a person, didn’t give unnecessary pushback, and was knowledgeable about my conditions (which I haven’t had in the past really) was amazing”

(DE; genderfluid, bisexual, pansexual, queer; Non-Hispanic White; Provider Requests).

In contrast, members also reported to CCs that clinical operations, providers, or staff did not meet expectations. For example, this member reported an incident that involved being misidentified:

**Member:** [E]ven after telling the person I initially called that I had a preferred name, the person who called to confirm my appointment still addressed me by my legal/dead name. … it was written down SOMEWHERE in their system, and I appreciated that they corrected their initial mistake. … Again, I’m sure their staff is very nice and it’s just a matter of getting around to updating forms and operating practices to accommodate for a (hopefully) increasingly inclusive society and culture.

**CC:** This super helpful – we can continue to recommend this space but activate for them to update their forms and be conscious of asking all patients the same questions around pronouns and preferred names.

(AZ; trans-feminine, genderfluid, lesbian and other; no race/ethnicity data; Provider Request)

Using member feedback, some providers were removed from the directory due to a lack of quality care delivery to LGTBQ+ patients OR not meeting criteria in connection with patient visits. Thus, CCs worked to expand (or contract as needed) its database of GAC appropriate legal, medical, and mental health providers for whom they could vouch. Members relied heavily upon this aspect of the healthcare navigation service. The excerpt below illustrates a common approach to discussing concurrent vetting practices of providers with whom members have not yet had direct interaction:

**CC**: I have identified the following providers who offer gender-affirming vocal coaching and speech therapy. Although we have not had direct member experience with all the providers below, I called each office personally and spoke to them to understand their practice, comfort, and experience working with the LGBTQ+ community. I can confirm they meet our vetting criteria, and we feel confident they are friendly and affirming.”

(CO, Transgender Woman, Non-Hispanic White, Speech Therapist request)

The exchange below illustrates member preferences related to provider characteristics and the CC’s confirmation that they would begin to evaluate providers in the area to ensure the member worked with someone well suited to his needs.

**Member** Since this will be the next step in gender-affirming surgery it would be good to have someone that has experience in this area. After this surgery there will be a plastic surgeon involved in final phase of surgeries.

**CC:** That is very understandable! I will start vetting potential options that are experienced in gender-affirming care/hysterectomies and update you on my progress.

(TX; transgender man, no sexual orientation information; Non-Hispanic, White; Provider Request, Resource Support)

Additionally, chats addressed whether providers could accommodate preferences for specific top and bottom surgical protocols, surgical revisions, and which providers practiced specified techniques. These discussions, while not enacting decision-aid tools directly, helped members filter for providers who performed techniques and offered services they needed.

#### Navigating complex insurance systems.

Navigating insurance systems is difficult for most people in the United States given the many complexities involved in accessing care, understanding polices, and the magnitude of uncertainties related to bills and payment structures. While in the prior theme we explore the ways in which CCs help TGD to navigate complex healthcare systems in terms of connections to competent and affirming clinicians, this theme focuses specifically on insurance complexities. System level inequities and barriers to care introduce added complexities for TGD individuals, together with their families, who want to address gender dysphoria by accessing GAC. This theme reflects solutions to systemic challenges that the navigation service (IH), CCs, and members co-created to together determine how to use resources to meet gender affirmation needs across the care continuum. These needs included care in the domains of medical-surgical affirmation, legal affirmation, psychological affirmation, and social affirmation. Three subthemes elucidate this system level finding: 1) access to accurate information about insurance policies and provider requests to facilitate meeting criteria for medical-surgical interventions, 2) assistance with appeals processes, and 3) obtaining letters of support. Below we will define each subtheme, and explore its importance to the larger theme, *Navigating Complex Insurance Systems*, using quotes from chat exchanges to highlight the significance of each.

#### Accessing accurate information about insurance coverage.

This sub-theme involves the process of accessing accurate information about insurance policies to facilitate meeting criteria for biomedical interventions for gender affirmation. Through CCs, members gained access to important information about GAC. CCs linked members with curated member resources, details about insurance policy coverage, and information about criteria needed to fulfill coverage requirements. Additionally, they supplied members with critical information to help with billing challenges and claims processes.

Information seeking, acquisition of verified information, and information use can feel daunting and overwhelming in complex healthcare contexts as evidenced by this member: “Awesome. Thank you so much. I have been so overwhelmed with just finding out what’s covered and what not...” (MO; queer, transgender woman; Non-Hispanic, White; Provider Requests). Further, member populations are not accustomed to navigating gender-affirming insurance policies, as insurance companies have only recently begun to incorporate transgender-specific offerings. Thus, inquiries like this one were seen repeatedly in the data: “Is there a way to know what my insurance covers for gender reaffirming surgery? I have never had insurance that covered these things, so it is all new to me in terms of finding care and what is covered and things like that.” (TX; transgender woman, bisexual, pansexual; African American, Hispanic Latinx, non-Hispanic White; Resource request for trans coverage; Provider Request). Support with information navigation through chat conversations included direction about what gender-specific policies included and the conditions members needed to fulfill for their care to be covered.

In another instance, the CC clarifies health plan details for the member who obtained some misinformation from a colleague: “there was some misinformation regarding your plan coverage that they’re in the process of updating and correcting…Once I’ve compiled all of the conflicting information, I will be sure to send it over,” (DE; genderfluid, bisexual, pansexual, queer; Non-Hispanic, White; Provider Requests). This exemplifies how CCs found and disseminated accurate information about GAC health plan features.

#### Assistance with appeals processes.

Many chats focused on issues related to appeals procedures, which suggests the breadth of CC and IH support with accessing care. That is, the care members seek is not unidimensional, which suggests the importance of CCs’ navigation expertise working between providers, insurers/payors, and patients to facilitate use of crucial interventions.

Member: Also, I just received the determination from [INSURER] on my appeal … and they denied it, citing the … pricing as the reason even though they are not in compliance with that term.

CC: Thank you for sharing that update! I am disappointed to learn that [INSURER] denied your first appeal... Since appeals can be time sensitive, it would be helpful to submit this form sooner than later…After you submit your form, I will have authorization to call [INSURER] on your behalf…. Although your appeal was denied, we can submit a second appeal to [Insurer].

(OR; No SOGI or race/ethnicity data; Provider Requests)

This appeal dealt with a gender-affirming surgical procedure for which the member had prior authorization. The member explains:

Member: They did approve a benefit level exception for the surgery as well, so it was processed at an in-network rate. I think my main concern is that the “allowed amount” seems extremely [REDACTED] -- about [REDACTED] for bottom surgery. While they wouldn’t tell me what amount they contract with in-network providers for or how that number is determined, my understanding is that this surgery costs significantly more in most cases with the average cost being about $26,000 understanding is that the “allowed amount” is what the insurance company has deemed to be a fair price for services, which does not seem to be the case here given the information I have. …

CC: I am glad you were approved for coverage at the in-network rate! I will definitely take a closer look at how your reimbursement was processed and try to figure out what potential options we have moving forward. In order to do this, I have a few questions:

1) When you got this in-network benefits approval, did they mention what the allowed amount was at the time or is this something you just recently learned about?
2) Have you or your surgeon received any written documentation regarding your approval for the exception?...

Unsurprisingly, chat conversations focused heavily on insurance coverage, with appeals coverage highlighting some of the strengths of a healthcare navigation services’ support for TGD.

#### Obtain letters of support to receive care.

The subtheme *obtaining letters of support to receive care* reflects issues at the intersection of SGM Care guidelines and structural stigma [30] (e.g., state, insurer, and health system policies, combined with institutional practices). WPATH Standards of Care 8 (SOC8) [3] supplies clinical guidance with the intention of optimizing, rather than impeding, gender-related care for TGD individuals. Members in our sample inquired with CCs about how to obtain letters as they initiated new GAC, maintained continuity of care (e.g., primary care or therapy), or wanted to take additional biomedical or surgical steps. Both clinical providers and insurance companies codify these letters of reference in policies required by patients prior to accessing care. Chat conversations reflected the multiplicity of policies and requirements related to letters of support, which varied by insurer, policy, and provider even if a patient sought the same intervention. For example, this CC acknowledged that the acquisition of letters can be confusing given that “requirements change all the time and are specific to your employer’s policy and plan.” Consequently, CCs helped many members find professionals who could provide such letters in a timely fashion. However, some encountered long wait times to obtain these assessments and associated letters for several reasons. First, and most notably, CCs mentioned increasing wait times, especially in “large medical centers,” where the uptick in people seeking GAC has created a situation in which increased demand and stable supply leads to longer than usual wait times for appointments with qualified providers. Second, CCs and members noted the increased difficulty of finding qualified providers in several states given the uncertainty created by legislative actions meant to limit access to GAC. Last, members commented on the need for financially feasible paths to acquiring letters of support from qualified mental health providers. Some members sought out free or low-cost, one-time consults through which to obtain letters of support because of limited resources. Reflecting the need for cost flexibility among TGD seeking letters of support, one care coordinator shared information “From the [provider’s] website regarding price: “I do these evals on a pay-what-you-can basis, so you decide what fits your budget.”

## Discussion

To our knowledge, this is the first study to explore the role and influence of virtual navigation for those seeking GAC by applying qualitative methods to redacted chats between a national sample of commercially insured individuals in the United States and LGBTQ+ focused CCs. We found that CCs influence members’ ability to successfully access identity-affirming care reflected in three themes: *Providing A Dignified Member Experience, Promoting Affirming Patient-Provider Relationships with Vetted Providers, Navigating Complex Insurance Systems*.

Three themes align with and build upon prior research on the importance of specialized patient navigation for TGD individuals [10]. First, *Providing a Dignified Experience,* specifically through the subtheme, *Shared life experience; no need to explain oneself*, demonstrates members’ perceptions of the value of training patient navigators (who also have LGBTQ+ community affiliations) to work with TGD individuals [31]. The second and third themes, *Promote Affirming Patient-Provider Relationships and Competent Care* and *Navigating Complex Insurance Systems* aligns with results from prior studies’ that found navigators improved TGD patients’ abilities to navigate multidimensional, intersecting insurance and healthcare systems [32] and that navigators were critical to accessing care among individuals seeking gender affirming surgeries [10]. Both *Navigating Complex Systems* and *Connections with Vetted Providers* also demonstrate members’ conceptualization of CCs as both advocates and administrative support, necessary to obtain GAC and navigate systems [31]. In that sense, CCs also occupy rich social roles accompanying TGD people in health-related journeys, which has implications for patient-provider relationships.

Our findings underscore the significant implications of the role and impact of healthcare navigation services, which represent an organizational manifestation of recognition and inclusion of TGD people, and reinterpretation of institutional norms. Further, the health navigation organization impacts individual, interpersonal, and structural levels, which aligns with the National Institute of Minority Health and Health Disparities’ theoretical framework adapted to address to Sexual and Gender Minority populations [33]. First, at the individual level, the theme *Providing a Dignified Experience* has several implications. For TGD and CCs, social interactions through chats reflects shared identity construction and meaning making. Second, at the interpersonal level, *Promoting Affirming Patient-Provider Connections*, suggests the importance of interventions that contribute to sustainable, affirming relationships that encourage quality care and outcomes. Third, at the organizational level, the structure of the navigation organization facilitates provision of respectful, dignified care to historically marginalized and stigmatized groups who often did not believe they could or would find help. This suggests that organizational processes can be reframed to affirm people’s identities and inherent dignity.

Like previously published studies, our data showed that many TGD members, particularly in certain regions of the U.S., faced long wait times as they sought care with affirming providers [3,8,34,35]. Similar to previous work, we also suggest that that pairing TGD individuals with vetted and competent providers and staff, who promote positive connections, may enhance care experiences, promote engagement, and improve outcomes [3,4,31,36]. Finally, the multidimensional roles and impact of trained, inclusive CCs has systemic implications. This is particularly true for TGD individuals who face systemic barriers such as high costs and difficulty navigating complex systems [37,38]. Currently, while some in the U.S. may have access to healthcare navigation services through employer benefits programs, fewer have LGBTQ+ specialized navigation. We agree with other authors who suggest that scalable, system level changes that incorporate navigation service reimbursement are needed to reach a greater proportion of the TGD population [5,37,39]. The recent Centers for Medicare & Medicaid Services (CMS) policy change that permits Medicare payments for navigation services [30], is a start that may promote healthcare access and improve outcomes for a larger portion of the population.

### Strengths and limitations

This study has several strengths. First, it includes chat data from a sample of over 460 commercially insured individuals seeking GAC from across all four census regions of the United States. Second, this is the first study, to our knowledge, that analyzes unique chat data between CCs and TGD individuals or their family members. The distinctive chat dataset enabled us to identify and explore the role and influence of CCs on members’ access to care without potential biases often present in qualitative studies reliant on interviews or focus groups, such as recall or agreement bias. Additionally, while our findings demonstrate that commercially insured individuals who have access to healthcare navigation services tailored to their needs benefit at multiple levels, virtual navigation services like these are scalable. Scaling a healthcare navigation intervention tailored to TGD and their families may have a measurable impact on healthcare costs and health outcomes and is a worthwhile step for future research.

However, several limitations also apply to this study. First, the chats between care coordinators and TGD patients were extensively redacted to protect PHI and PII, making it possible that nuances in some conversations were made opaque, resulting in analysts potentially missing relevant insights. We minimized this limitation by having multiple coders, coder debates and discussions that leveraged diverse experiences and perspectives, and efforts to reach consensus in theme development. Second, while we had a substantial volume of chat data, limited resources and time for analysis led us to analyze randomly selected chats. Not being able to include all data is never desirable. However, our study sample of over 460 members is large for a qualitative study (particularly compared to those that employ interview or focus group data collection methods). As a result of the sample size and dyadic nature of the conversation data, this study offers unique insights based on candid interactions that convey insights about the usefulness of health navigation for TGD. Third, given the data structure where multiple chat records can be related to the same case, the analysis may have been biased due to the potential inclusion of multiple chats from the same case. Fourth, since the data was not originally collected for research purposes, we lacked information on the specific nature of each member’s insurance benefits or the full range of employer-based benefits they had access to (e.g., types of insurance products, insurance policies, and benefits packages). This posed challenges in matching navigator requests to specific available resources. Similarly, information on members, such as income or education level were not available. This absence of data limits our ability to fully understand the study sample, reach naturalistic generalizability of the results [40–42] results, or stratify the results by member subgroups. Last, the chat data was limited to TGD individuals with commercial insurance and access to virtual healthcare and health navigation support. As such, the experiences reflected in the data and findings are specific to those with Included Health and the navigation services members accessed. Thus, our findings may not reflect the experiences of individuals without commercial insurance, those with public insurance, or TGD to who do not have health navigation support.

## Conclusion

This study has generated evidence that suggests the importance of the role and positive impact of trained CCs for TGD populations who seek GAC. Not only do trained CCs help to cultivate dignified member experiences for those who historically face stigma and marginalization, they help to connect members with competent and affirming providers and assist with navigating complex and fragmented systems. These findings reflect the multiple levels of influence rendered through a tailored navigation service, aligning with the NIMHD’s Sexual and Gender Minority framework, which focuses on reducing health disparities and contributing to improved access and care for all. At the levels of individuals, interpersonal, and structures, healthcare navigation services may contribute to promoting improved access to affirming providers and potential health benefits. While our findings demonstrate that commercially insured individuals who have access to healthcare navigation services tailored to their needs benefit at multiple levels, virtual navigation services like these are scalable. Scaling a healthcare navigation intervention tailored to TGD and their families may have a measurable impact on healthcare costs and health outcomes and is a worthwhile step for future research.

## Supporting information

S1 Data Demographic Data. (CSV)
